# Expired EBT3 Films’ Sensitivity for the Measurement of X-ray and UV Radiation: An Optical Analysis

**DOI:** 10.3390/ma15082903

**Published:** 2022-04-15

**Authors:** Hamad Yahia Abu Mhanna, Ahmad Fairuz Omar, Yasmin Md Radzi, Hanan Fawaz Akhdar, Haytham Al Ewaidat

**Affiliations:** 1 School of Physics, Universiti Sains Malaysia, Penang 11800, Malaysia; yasminradzi@usm.my; 2 Physics Department, College of Science, Imam Mohammad Ibn Saud Islamic University (IMSIU), Riyadh 13318, Saudi Arabia; 3 Department of Allied Medical Sciences-Radiologic Technology, Faculty of Applied Medical Sciences, Jordan University of Science and Technology, Irbid 22110, Jordan; haewaidat@just.edu.jo

**Keywords:** colorimetry, EBT3, expiry date, visible spectroscopy

## Abstract

The aim of this study is to compare the optical responses of external beam therapy 3 (EBT3) films exposed to X-rays and solar ultraviolet rays (SUV-rays), as a dose control technique in the clinical sector for various radiation types, energies, and absorbed doses up to 4 Gy. In this study, EBT3 films with three different expiry dates were prepared and cut into pieces of size 2 by 2 cm^2^. The first group was exposed to 90 kVp X-rays, while the second group was exposed to the SUV-rays at noon. The analysis was performed using a visible Jaz spectrometer and an EPSON Perfection V370 Photo scanner to obtain the absorbance, the net reflective optical density (ROD) and the red-green-blue (RGB) values of the samples. The results have shown that spectroscopic measurements of the exposed expired EBT3 films with these radiation sources are able to produce primary peaks and secondary peaks at λ = 641.74 nm and λ = 585.98 nm for X-rays, and at λ = 637.93 nm and λ = 584.45 nm for SUV-rays, respectively. According to these findings, compared to 2021 films that expired shortly before the trial start date; 2018 films responded better to the absorbed dose than 2016 films when exposed to both X-ray and SUV-rays. In terms of energy dependence, the expired EBT3 2018 had the largest net ROD value. Using L*a*b* indices extracted from the RGB data, and despite that EBT3 films have expiry dates according to the manufacturer; all the films exhibited a substantial colour change, indicating that these films are still usable for clinical and research purposes.

## 1. Introduction

The first Gafchromic™ EBT films were launched for dosage verifications and doses assessments in external beam treatments more than a decade ago [1,2]. Because of their high spatial resolution, radiochromic films are widely used in radiology and radiation treatments, and quality assurances (QA) [3,4,5,6,7]. They can also be submerged in water and cut, with no requirement for chemical processing, they also have low sensitivity to visible light [8,9]. The active component of radiochromic films is diacetylene monomers, which polymerise when exposed to light. As more polymerisation is absorbed, the film darkens [10,11,12]. Recently, the newest EBT3 model Gafchromic™ film was launched. According to the manufacturer International Specialty Products, EBT3 films are constructed similarly to EBT2 films and exhibit similar expected performance characteristics, and it is a radiation-sensitive film made up of an active layer sandwiched between two matte-polyester substrates [13]. The active layer comprises a marking dye, a stabilising agent, and the active component, the diacetylene monomers, all of which contribute to the films’ near-perfect energy independence (5% at 100 keV and 18 MeV) [14]. The active component undergoes a 1,4-polymerisation process after being exposed to ionising radiation or thermal annealing, which changes the optical density depending on the active layer’s composition [15,16], which produces an absorption peak at (420 nm) that can be used to compensate for differences in the sensitive layer thickness, but with the addition of a symmetrical and an anti-Newton ring artefacts coating [17,18,19]. EBT3 Radiochromic film is an extremely useful device for both relative dosimetry (measuring the properties of the radiation field) and reference dosimetry. Radiochromic films are used in advanced radiation therapies (e.g., stereotactic radiosurgeries (SRS), and intensity modulated radiotherapies (IMRT) [10,20]. Due to their obvious energy independence, great spatial resolutions, near tissue equivalencies, and ease of handling, EBT3 GafChromic™ films are ideal instruments for 2D dose assessments. With increasing dosage, the films darken, indicating a reduction in optical transparency or an increase in optical absorption [21]. Due to their high ionising radiation sensitivity [22], these films are polymerised when ionising and UVR are exposed, which results in changes in the absorber characteristics of the visible range of the films. These films are not visible light sensitive and may be handled and processed under room light. Many studies have evaluated the sensitivity of radiochrome films to UVR to assess changes in their optical densities (ODs) [23,24]. Few studies have used UVR dosimetry of these films for their wide-band UVB and UVA reactions [19]. In practise, a flat-bed scanner is used to evaluate the diminishing transparency of EBT3 GafChromic™ films. The values of the scanned RGB pixels may be utilised to quantitatively estimate the radiation dosage [21]. When EBT3 films are exposed to UV light and their colour begins to change from light green to dark green, they are regarded to have reached their expiration date. This is due to the fact that when exposed to UV radiation, the films can no longer be used. Unexposed films must be kept in a dark environment at room temperature (20–25 °C) and away from any sources of radiation. When kept at normal temperature, the film has a two-and-a-half-year shelf-life. An EBT3 film’s performance should not be affected by a brief exposure to 70 °C or a longer exposure of one day at 50 °C [12,25]. In this study, expired films were utilised to demonstrate the usefulness of these films after the expiration dates based on the manufacturer and their compatibility with all available evidence and research on the validity of using items after the expiration dates. In practise, any perishable item deteriorates (or degrades) with time, reaching 100% by the expiration date. Failure to consider the effect of the expiration date on demand may result in a biased solution. Researchers also emphasise the importance of expiration dates, such as for retailing and consumer research, as consumers take this product characteristic into account when making purchase and consumption decisions about perishables [26,27]. An expiry date is a set period after which something should no longer be sold or utilised due to a predicted deterioration in quality or effectiveness. There are no federal rules specifying what different types of expiration dates represent or forcing manufacturers to include appropriate expiration date information on product packaging. Furthermore, various academics in this field are investigating how expiry date-based pricing works [28,29,30].

EBT3 was developed for the purpose of determining the absorbed doses of ionising and non-ionising radiation. Following the EBT3 film’s exposure to radiation, it will progressively alter its hue, by ionising and non-ionising radiation, from bright green to darker [15]. This study focuses on investigating the efficacy of the expired EBT3 films and the optical responses of the expired EBT3 films measured using visible spectroscopy and net reflective optical density (Net ROD). Moreover, analysis was performed on the films to characterise the dosimetric properties of X-rays and SUV radiation and the possibility of using the coloration of expired EBT3 films measured using visible spectroscopy to develop X-ray and SUV-rays and the dosimetric properties in future studies. We demonstrate the effectiveness of these films when exposed to radiation even after their expiration dates, which indicates that they can be stored and used well when needed, compared to non-expired films.

## 2. Materials and Methods

### 2.1. Radiography X-rays Experiment Procedure

GAFChromic™ EBT3 films were classified into three groups according to their expiration dates: group A (expired in July 2016, Batch No 07291401), group B (expired in March 2018, Batch No 03021601), and group C (expired in May 2021, Batch No 05161903). These films were cut into 33 identical 2 cm by 2 cm squares; 33 black envelopes were prepared to protect the films from scratches, and pollutants and to keep them out of any light exposure. The above measurements shall include 11 films for each of the categories, which shall be subdivided into two groups, each with five films in a single group and one film used as a reference. To start, the Toshiba Radrex KXO-50S general X-ray radiography apparatus was heated at 90 kVA, 100 mAs, 10 cm × 10 cm, and a 40 cm SSD setup. Then a semiconductor radiation detector was connected to an electrometer, and one piece of an EBT3 film sample was placed alongside the PTW semiconductor dosimeter for diagnostic radiology in the center of equal field size. The experiment was conducted on 15 films prepared in advance. First, we chose the EBT3 film collection that expired in 2016, consisting of five films. Irradiation was performed on the film sample up to the desired accumulated radiation dose in the range of (0.5 Gy–4.0 Gy). After the process is completed, the film samples were placed inside black envelopes to avoid any contamination from ambient light. Then, the steps were repeated for four more samples of films that expired in July 2016. The previous experience was also repeated for EBT3 films that expired in March 2018 and May 2021.

### 2.2. Solar Exposure on EBT3 Film

The other fifteen films were exposed to sunlight at the same time, place and at the same level. The operation was carried out on (27 June 2021) at (1:13 p.m.), (33.4–35.2 °C) (pressure 54–60%) (humidity 55.2) at the School of Physics, Universiti Sains Malaysia. The building’s rooftop was chosen as the experimental location to ensure the maximum exposure to sun irradiance towards the surface of the films. During the experiment, the weather was hot with the on and off appearance of clouds. All films were exposed to sunlight at the same time, place, and conditions. The UV meters were positioned adjacent to the EBT3 films during the measurement to determine the total exposure on each EBT3 film and the thermometer. The experiment was set up in such a manner that there would be 15 films with an incremental level of film discolouration or an incremental level of UV doses. After sun exposure, every 3 min, one exposed film was collected from each group to obtain the accumulated UV dose levels, then the exposed films were placed inside black envelopes to prevent further exposure to UV rays. UVA-UVB radiation was measured in mW/cm^2^ every minute using UVA and UVB radio counters, models 4.0 and 6.0, respectively. UV dose estimates for films in mJ/cm^2^ were calculated by multiplying the total irradiance of UVA and UVB by the exposure time. During the experiment, the procedure was completed in 15 min.

### 2.3. Absorbance Spectroscopy Measurement

After completing both experiments, the films’ absorbances were determined at the Engineering Physics lab at Universiti Sains Malaysia’s School of Physics using an Ocean Optics Jaz spectrometer. The spectrometer operates at wavelengths ranging from 200 to 1100 nm (Ocean Optics, Inc., Dunedin, FL, USA). A tungsten halogen lamp (HL-2000) with spectral emission between 360 and 2500 nm and a colour temperature of 2960 K was used in the spectroscopy system, the Jaz spectrometer was attached to a tungsten halogen lamp via a cuvette holder and an attenuator via optical fibre cables. In the cuvette holder, the EBT3 films were mounted. The attenuator’s job is to minimise or raise the strength of halogen lamp light in unit counts. Before beginning the measurement, the spectrometer was turned on, allowing the lamps to heat up for a suitable period of time to steady them. The performance was shown using Spectrasuite software on the computer panel. Spectrasuite is a spectroscopy application that is used to collect and analyse experimental spectrum data. A non-exposed film was used to calibrate the device. As a reference, an unexposed EBT3 film was utilised, and the EBT3 film was put in a CUV 1 cm Cuvette Holder for this absorbance spectroscopy experiment. Scan time, average time, and boxcar width were set at 3 ms, 30 ms, and 1, respectively. After setting the important configurations, a card was slit through a tungsten halogen lamp to avoid light from passing through and to reduce the dark current in the software. To enable the light to pass through again, the card was removed from the tungsten halogen lamp. The program was set to absorbance with the letter ‘A,’ and the performance indicated 0 absorbance for the unexposed film. Following the calibration procedure, each sample of the exposed film was subjected to an absorbance study. The performance absorbance calculation for each exposed film was saved. The best wavelength was determined depending on the maximum peak absorption range, and data was collected.

All expired EBT3 films (exposed to X-ray and SUV-rays) were scanned using an Epson scanner. The scan configuration was set at 600 dpi for 48-bit optical resolution and image color. The scanned image was saved in TIFF format. ImageJ software was used to analyse the scanned picture. Filtering by the red channel was chosen. The mean grey value for a single piece of film was determined by taking into consideration the standard deviation of the mean grey value.

## 3. Results and Discussion

**Figure 1:**
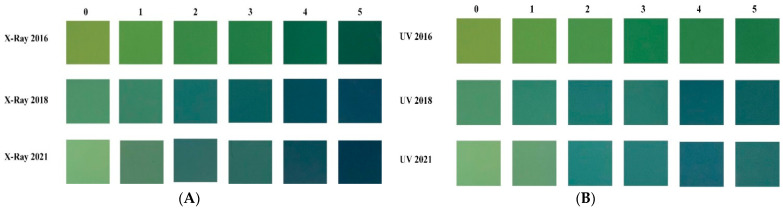
Incremental changes to the discoloration of expired EBT3 film for X-ray (**A**). Incremental changes to the discoloration of expired EBT3 film for SUV (**B**).

As shown in Figure 1, the increase in the degree of discolouration on expired EBT3 films is strongly related to the amount of X-ray and SUV-rays exposure on the films. The image depicts the film before X-ray and SUV-rays exposure (leftmost) and the progressive change in colour of the film after incremental X-ray and SUV dosage exposure. According to the observation, the film’s initial hue (light green) darkens (dark blue).

The radiation-induced polymerisation process of a lithium salt of pentacosa 10,12-diynoic acid is the mechanism behind the film’s decreasing transparency (LiPCDA) [15]. Because of the two conjugate triple carbon-carbon bonds towards the centre of the hydrocarbon chain, PCDA monomers are colourless, long-chained molecules that belong to the diacetylenes (DA) class [31,32,33]. LiPCDA monomers are organised in microcrystals that are scattered in the film’s active layer (usually 28 m). The monomers can polymerise and produce a long-chained polymer (poly-PCDA) with alternating double and triple carbon-carbon bonds in the backbone in this arrangement. Because of the substantial delocalisation of -orbital electrons, the backbone absorbs light in the visible range. The poly PCDA concentration rises as the dosage rises [12,34].

**Figure 2:**
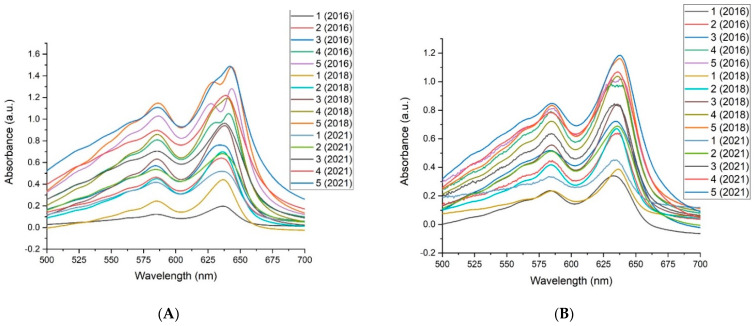
Visible absorbance spectra for EBT3 films irradiated by 90 kVp X-rays (**A**). Visible absorbance spectra for EBT3 films by SUV (**B**).

Figure 2 illustrates the net visible light absorption spectrum (optical density) of expired EBT3 films when subjected to X-ray and solar UV doses. The net absorption spectra of the films are visible in these figures as peak absorption bands, the greatest (primary) values of X-rays were focused around 641.74 nm, while the lowest (secondary) values of X-rays were centred around 585.98 nm. In contrast, the greatest (primary) values of SUV-rays were centered around 637.93 nm, while the lowest (secondary) values of SUV-rays were centred around 584.45 nm.

From the obtained results, the core peak band at λ = 641.74 nm, all primary absorption bands lie around the 641.74 nm wavelength at each absorbed dose. With increasing the absorbed doses, the value of peak absorption also increases. For the 90 kVp X-ray experiment, the spectra indicate 1.488 as the highest absorption value at the 3.939 Gy absorbed dose. For the SUV experiment, the absorbed dose of 1.186 was the highest absorption value. Therefore, expired EBT3 films show a high response to X Rays compared to the other experiments. These results of the absorbed dosage indicate that expired EBT3 films from 2018 reacted better than films from 2016, compared to films of 2021 that expired shortly before the experiment’s starting date. Both experiments yielded identical results (X-ray and SUV-rays). This implies that as the expiration date increases, the absorbance of films gradually decreases, as shown by comparison to expired EBT3 films from the year 2021.

The higher R^2^ (coefficient of determination, or the coefficient of multiple determination for multiple regression) shows how close the data are to the fitted regression line. As the value of R^2^ approaches the value of 1 or a percentage of 100 percent, the closer the data is to the fitted regression line or the accuracy of the reading. The line slope indicates the reaction of expired EBT3 colour toward radiation exposure. The greater slope implies higher responsivity. This responsivity changes from one wavelength to the other based on the transition range of expired EBT3 colours.

**Figure 3:**
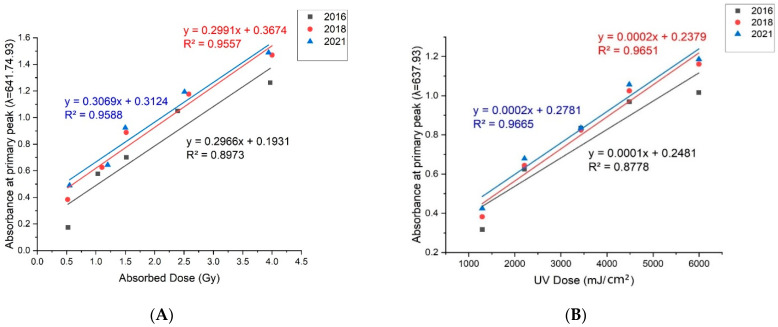
Linear graph of absorbance of primary peak at λ = 641.74 nm (X-ray) (**A**). Linear graph of absorbance of primary peak at λ = 637.93 nm (SUV) (**B**).

From the linear regression analysis of the results obtained for X-ray, in the base peak range at λ = 641.74 nm, the 2021 expired EBT3 films showed higher responses than the other films with a regression value of m = 0.3069 and showed the best fit regression line with the highest value of R^2^ for others who scored 95.9%. The 2016 expired EBT3 films showed the lowest response with a regression value of m = 0.2966 and the lowest regression level of 89.7%. Therefore, the 2021 expired EBT3 films show higher radiative responses than the other films as shown in Figure 3A. Concerning the SUV findings achieved, in the base peak range at λ = 637.93 nm, the 2021 expired EBT3 films showed higher responses than the other films with a regression value, m = 0.0002 and showed the best fit regression line with the highest value of R^2^ for others which scored 96.7%. The 2016 expired EBT3 films showed the lowest responses with a regression value of m = 0.0001, and the lowest regression level of 87.8%. Therefore, the 2021 expired EBT3 films show higher SUV responses than the other films. as shown in Figure 3B.

The mean pixel value (MPV) for each expired EBT3 film was determined using ImageJ software and the net ROD is computed using the Equation (1). The findings are summarised in the table below. The net ROD value for each absorbed dose from two distinct sources of radiation is shown in Table 1.

(1) $$\mathrm{Net}\;\mathrm{ROD}=\log\;(p_{u}/p_{t})$$

where *p_u_* denotes the pixel value of the reflected intensity via an unexposed film at the orientation in which the maximum pixel value is obtained, and *p_t_* denotes the pixel value of the reflected intensity at any other film orientation or irradiation level [34,35].

In terms of the energy dependence of the X-ray experiment, the results showed that the expired EBT3 2018 exhibited the highest net ROD value for X-ray radiation from the 2016 expired EBT3, compared with the expired EBT3 2021. For example, at an absorbed dose close to 1 Gy, the year 2018 showed the highest net ROD value of 0.108 from 2016 which got a net ROD of 0.102 compared to 2021 which had a net ROD value of 0.123, As shown in Table 1A. In terms of energy dependence in the SUV experience, the expired EBT3 2016 also appears to show the highest net ROD value, compared with the expired EBT3 2021. For example, at an absorbed dose close to 5994 mJ/cm^2^, 2018 showed the highest net ROD value of 0.068 from 2016 which had a net ROD of 0.147 compared to 2021 which had a net ROD value of 0.187, As shown in Table 1B.

The film pictures are presented in Figure 1. These pictures were saved in the JPEG format, and the images were analysed using the free software Get Average Color of Image, which may be found at https://matkl.github.io/average-color/ (accessed on 30 June 2021) to obtain the films’ Red-Green-Blue (RGB) colour values. Using the colour distribution of each expired EBT3 film, the RGB value representing each film was calculated using the image’s average RGB values. The RGB data for each film was then transformed to the CIE 1976 L*a*b* colour space (a.k.a. CIELAB) using the ColorMine online converter, which is publicly accessible at http://colormine.org/convert/rgb-to-lab (accessed on 30 June 2021). Some academics have made extensive use of ColorMine to convert colours from RGB to L*a*b* and vice versa.

Figure 1 shows the final images of the films after exposure to X-rays and SUV-rays. It seems that the overall appearance of the color varies as the image on the right appears to be lighter than the last image of the film on the left, which appears in a darker color. The color appearance and color conversion of films were developed from a lower to a higher degree by analysing the CIELAB color representation of films.

**Figure 4:**
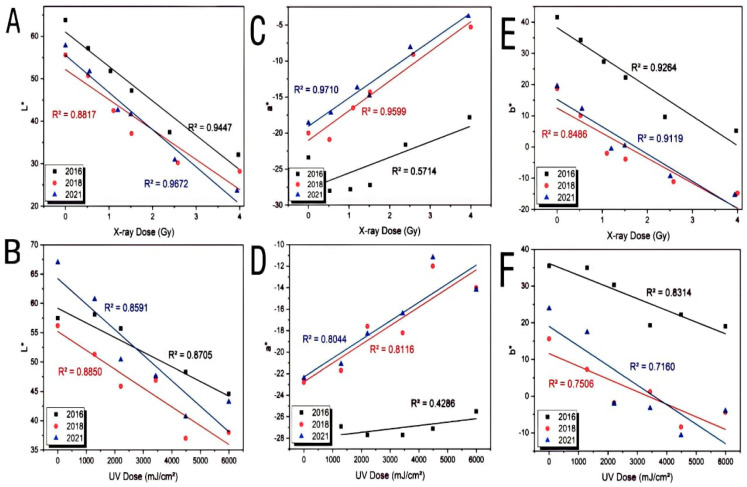
Shows the relationship between L*a*b and the degree of color effect. L* = lightness (**A**,**B**). a* = green-red (**C**,**D**). b* = yellow (**E**,**F**).

Figure 4 shows the relationship between L*a*b and the degree of color effect, where (A, B) shows the relationship between L* (lightness) and the color effect degree of (2016, 2018 and 2021) films exposed to X-rays and SUV-rays. In general, the L* values of 2016 films ranged between 32.1 and 63.8, while the values of 2018 films ranged between 28.2 and 55.6, finally, the values of 2021 films ranged between 23.6 and 57.8 for X-rays with an overall tendency toward the lower L* for higher color effect degree, indicating darkening in films. The values of L* for 2016 films ranged between 44.6 and 57.5, while the values of 2018 films ranged between 38 and 56.2, and finally, the values of 2021 films ranged between 43.2 and 67 for SUV-rays with an overall tendency towards the lower L* for higher color effect degree, indicating darkening in films. The graph in (C, D) depicts the relationship between the value of a* and X-ray Dose (Gy), and UV Dose (mJ/cm^2^) for SUV. The negative a* axis represents the green characteristics of films, while the positive a* axis represents the red properties of films. All films had negative a* values ranging from −17.8 to −23.4 for 2016 films, −5.3 to −20 for 2018 films, and lastly −3.8 to −18.6 for 2021 films for X-ray. The values of a* for 2016 films ranged between −25.5 and −17.3, and the values of 2018 films ranged between −14 and −22.8, and finally, the values of 2021 films ranged between −14.2 and −22.4 for SUV. These findings corroborated the presence of prominent green elements in all pictures. The yellow component (represented by b*) of the EBT3 films and its relationship with X-ray Dose (Gy), and UV Dose (mJ/cm^2^) for SUV is shown in (E, F). For 2016 EBT3 films, b* is at 5.2 as the yellow component of films starts to appear at higher absorbance, with the maximum b* reaching 41.6. The relationship between the yellow component of 2016 films and its absorbance is highly linear with R^2^ = 0.9264. In contrast, 2018 films start from −14.8 to 18.7 and absorbance is linear with R^2^ = 0.8486, and 2021 films start from −15.4 to 19.5 with R^2^ = 0.9119 for X-rays. The values of b* for 2016 films ranged between −19 and 35.5, and the values of 2018 films ranged between −4.4 and −15.6, and finally, the values of 2021 films ranged between −4 and 23.9 for SUV-rays.

**Figure 5:**
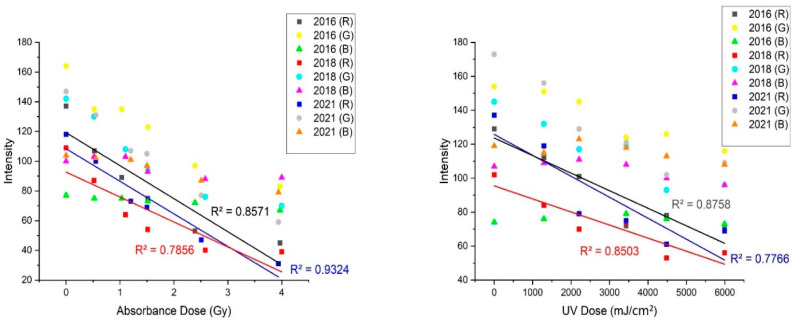
To RGB components for expired EBT3 films.

In this study, quantitative analysis changed the colors of films that had been exposed to X-rays and SUV-rays using the regression process with various RGB indices. Subscripts “2016”, “2018” and “2021” on the graph in Figure 5 refer to RGB components of the expired EBT3 films. Among individual R, G and B components, only R generated a good linear relationship with absorbance (Gy). Exponential regression between the components of the image (i.e., R 2021) produced a much higher prediction accuracy with R^2^ = 0.9324 for X-ray and (R 2016) with R^2^ = 0.8758 for SUV. The absorbance (Gy) and UV Dose (mJ/cm^2^) of the expired EBT3 films were inversely proportional to the intensity of the R component extracted from the images of the expired EBT3 films.

## 4. Conclusions

The study showed the suitability of expired EBT3 films for high-resolution SUV and X-ray dosimetry, as well as clinical applications. According to this study, it was found that the expired EBT3 film produces a change in its visible light absorption spectrum when irradiated with SUV and X-ray radiation. The degree of discoloration on expired EBT3 films increases in direct proportion to the quantity of X-ray and SUV exposure on the films. The absorption peaks of 90 kVp X-rays observed in this study were 641.74 and 585.98 nm, respectively, whereas the SUV values were 637.93 and 584.45 nm. According to linear regression analysis, expired EBT3 films in 2021 showed greater responsiveness, whereas expired EBT3 films in 2016 exhibited decreased responsiveness. In terms of the energy dependence of the X-rays and SUV-rays experiments, the results showed that the expired EBT3 2018 exhibited the highest net ROD value for X-ray radiation from the 2016 expired EBT3, compared with the 2021 expired EBT3. Among the R, G, and B components individually, only R had a strong linear relationship with absorbance (Gy).

## Figures and Tables

**Table 1:** (A) Net ROD value for radiography X-ray. (B) Net ROD value for radiography SUV.

(A)				
Source of Radiation	$\mathrm{Expired}\;\mathrm{EBT}3\;\mathrm{Film}\;\mathrm{Net}\;\mathrm{ROD}\;=\;\log\;(p_{u}$ $/p_{t})$ **Samples**	Dose (Gy)	Net ROD	RMSE (Gy)
**X-ray**	**EBT3 film (expired in July 2016)**	0.523	0.057	0.017
		1.032	0.102	
		1.517	0.145	
		2.391	0.229	
		3.969	0.289	
	**EBT3 film (expired in March 2018)**	0.519	0.043	0.032
		1.102	0.108	
		1.515	0.170	
		2.581	0.241	
		4.000	0.253	
	**EBT3 film (expired in May 2021)**	0.551	0.046	0.010
		1.200	0.123	
		1.500	0.132	
		2.508	0.241	
		3.939	0.347	
**(B)**				
**Source of Radiation**	**Expired EBT3 Film Samples**	**UV Dose (mJ/cm^2^)**	**Net ROD**	**RMSE (mJ/cm^2^)**
**SUV**	**EBT3 film (expired in July 2016)**	1292.40	0.043	0.028
		2210.40	0.098	
		3434.40	0.116	
		4485.60	0.121	
		5994.00	0.147	
	**EBT3 film (expired in March 2018)**	1292.40	0.032	0.025
		2210.40	0.061	
		3434.40	0.068	
		4485.60	0.152	
		5994.00	0.160	
	**EBT3 film (expired in May 2021)**	1292.40	0.043	0.015
		2210.40	0.107	
		3434.40	0.132	
		4485.60	0.169	
		5994.00	0.187	

## Data Availability

Not applicable.

## References

[1] Khachonkham S., Dreindl R., Heilemann G., Lechner W., Fuchs H., Palmans H., Georg D., Kuess P. (2018). Characteristic of EBT-XD and EBT3 radiochromic film dosimetry for photon and proton beams. Phys. Med. Biol..

[2] Aldelaijan S., Devic S. (2018). Comparison of dose response functions for EBT3 model GafChromic™ film dosimetry system. Phys. Med..

[3] Crijns W., Maes F., van der Heide U.A., Heuvel F.V.D. (2013). Calibrating page sized Gafchromic EBT3 films. Med. Phys..

[4] Bekerat H., Devic S., Deblois F., Singh K.K., Sarfehnia A., Seuntjens J., Shih S., Yu X., Lewis D.F. (2014). Improving the energy response of external beam therapy (EBT) GafChromic™ dosimetry films at low energies (≤100 keV). Med. Phys..

[5] Massillon J.L.G., Chiu-Tsao S.-T., Domingo-Munoz I., Chan M.F. (2012). Energy Dependence of the new Gafchromic EBT3 film: Dose response curves for 50 KV, 6 and 15 MV X-ray beams. Int. J. Radiat. Oncol. Biol. Phys..

[6] Das I.J. (2017). Radiochromic Film: Role and Applications in Radiation Dosimetry.

[7] Morales J.E., Butson M., Crowe S., Hill R., Trapp J. (2016). An experimental extrapolation technique using the Gafchromic EBT3 film for relative output factor measurements in small X-ray fields. Med. Phys..

[8] León-Marroquín E.Y., Lárraga-Gutiérrez J.M., Herrera-González J.A., Camacho-López M.A., Barajas J.E.V., García-Garduño O.A. (2018). Investigation of EBT3 radiochromic film’s response to humidity. J. Appl. Clin. Med. Phys..

[9] Méndez I., Rovira-Escutia J.J., Casar B. (2021). A protocol for accurate radiochromic film dosimetry using Radiochromic.com. Radiol. Oncol..

[10] Niroomand-Rad A., Chiu-Tsao S., Grams M.P., Lewis D.F., Soares C.G., Van Battum L.J., Das I.J., Trichter S., Kissick M.W., Massillon-Jl G. (2020). Report of AAPM Task Group 235 Radiochromic Film Dosimetry: An Update to TG-55. Med. Phys..

[11] Ahmad J. (2018). Development of Micrometer Resolution Dosimetry using Radiochromic Film by Raman Spectroscopy and Its Application to Measuring the Radioenhancement of Gold Nanofilm. Ph.D. Thesis.

[12] Osman U.S., Omar A.F. (2021). Visible Spectroscopy in EBT3 Solar Ultraviolet Dosimeter.

[13] Vadrucci M., Esposito G., Ronsivalle C., Cherubini R., Marracino F., Montereali R.M., Picardi L., Piccinini M., Pimpinella M., Vincenti M.A. (2015). Calibration of GafChromic EBT3 for absorbed dose measurements in 5 MeV proton beam and 60 Co γ-rays. Med. Phys..

[14] GafChromic™ Dosimetry Media, Type EBT-3. http://www.gafchromic.com/documents/EBT3_Specifications.pdf.

[15] Williams M., Metcalfe P. (2011). Radiochromic Film Dosimetry and its Applications in Radiotherapy. AIP Conference Proceedings.

[16] Watanabe Y., Patel G.N., Patel P. (2006). Evaluation of a new self-developing instant film for imaging and dosimetry. Radiat. Prot. Dosim..

[17] Sorriaux J., Kacperek A., Rossomme S., Lee J., Bertrand D., Vynckier S., Sterpin E. (2013). Evaluation of Gafchromic^®^ EBT3 films characteristics in therapy photon, electron and proton beams. Phys. Med..

[18] Gambarini G., Regazzoni V., Artuso E., Giove D., Mirandola A., Ciocca M. (2015). Measurements of 2D distributions of absorbed dose in protontherapy with Gafchromic EBT3 films. Appl. Radiat. Isot..

[19] Butson M., Cho G., Gill S., Pope D. (2017). Physics and characteristics of radiochromic films. Radiochromic Film.

[20] Billas I., Bouchard H., Oelfke U., Duane S. (2019). The effect of magnetic field strength on the response of Gafchromic EBT-3 film. Phys. Med. Biol..

[21] Callens M., Crijns W., Simons V., De Wolf I., Depuydt T., Maes F., Van Den Abeele K. (2016). A spectroscopic study of the chromatic properties of GafChromic™ EBT3 films. Med. Phys..

[22] Vaiano P., Consales M., Casolaro P., Campajola L., Fienga F., Di Capua F., Breglio G., Buontempo S., Cutolo A., Cusano A. (2019). A novel method for EBT3 Gafchromic films read-out at high dose levels. Phys. Med..

[23] Aydarous A., Al-Omary E.A., El Ghazaly M. (2014). Characterization of Gafchromic EBT3 films for ultraviolet radiation dosimetry. Radiat. Eff. Defects Solids.

[24] Reinstein L.E., Gluckman G.R., Amols H.I. (1997). Predicting optical densitometer response as a function of light source characteristics for radiochromic film dosimetry. Med. Phys..

[25] McLaughlin W.L., Al-Sheikhly M., Lewis D.F., Kovács A., Wojnárovits L. (1996). Radiochromic Solid-State Polymerization Reaction. ACS Symp. Ser..

[26] Wu J., Chang C.-T., Teng J.-T., Lai K.-K. (2017). Optimal order quantity and selling price over a product life cycle with deterioration rate linked to expiration date. Int. J. Prod. Econ..

[27] Theotokis A., Pramatari K., Tsiros M. (2012). Effects of Expiration Date-Based Pricing on Brand Image Perceptions. J. Retail..

[28] Tsiros M., Heilman C.M. (2005). The Effect of Expiration Dates and Perceived Risk on Purchasing Behavior in Grocery Store Perishable Categories. J. Mark..

[29] Hall-Phillips A., Shah P. (2017). Unclarity confusion and expiration date labels in the United States: A consumer perspective. J. Retail. Consum. Serv..

[30] Bar-Anan Y., Liberman N., Trope Y. (2006). The association between psychological distance and construal level: Evidence from an implicit association test. J. Exp. Psychol. Gen..

[31] Callens M.B., Crijns W., Depuydt T., Haustermans K., Maes F., D’Agostino E., Wevers M., Pfeiffer H., Abeele K.V.D. (2017). Modeling the dose dependence of the vis-absorption spectrum of EBT3 GafChromic™ films. Med. Phys..

[32] Mirza J.A., Park H., Ye S.-J. (2016). Use of radiochromic film as a high-spatial resolution dosimeter by Raman spectroscopy. Med. Phys..

[33] Lewis D., Micke A., Yu X., Chan M.F. (2012). An efficient protocol for radiochromic film dosimetry combining calibration and measurement in a single scan. Med. Phys..

[34] Butson E.T., Cheung T., Peter K.N., Butson M.J. (2010). Measuring solar UV radiation with EBT radiochromic film. Phys. Med. Biol..

[35] Chan P.M., Ng C.Y.P., Beni M.S., Law S.S., Yu K.N. (2017). Auto-development issue in quality assurance of biological X-ray irradiator using Gafchromic EBT3 film. Results Phys..

